# Selected hematological abnormalities and their associated factors among asthmatic patients in Northwest Ethiopia: a cross-sectional study

**DOI:** 10.1186/s12890-022-02020-z

**Published:** 2022-06-13

**Authors:** Yenealem Solomon, Berhanu Woldu, Nebiyu Mesfin, Bamlaku Enawgaw

**Affiliations:** 1 Department of Hematology and Immunohematology, School of Biomedical and Laboratory Sciences, University of Gondar, Gondar, Ethiopia; 2 Department of Medical Laboratory Science, College of Health Sciences, Debre Tabor University, Debre Tabor, Ethiopia; 3 Department of Internal Medicine, School of Medicine, University of Gondar, Gondar, Ethiopia

**Keywords:** Hematological abnormalities, Eosinophilia, Neutrophilia, Asthma, Leukocytosis

## Abstract

**Background:**

Asthma is a chronic inflammatory disease that affects the lungs. Variation in whole blood cell lines is caused by the progression and severity of asthma. Common hematological abnormalities encountered during asthma include eosinophilia, neutrophilia, leukocytosis, and increased erythrocyte sedimentation rate. The main aim of this study was to assess the selected hematological abnormalities and their associated factors among asthmatic patients in Northwest Ethiopia from March to May 2021.

**Methodology:**

A hospital-based cross-sectional study was conducted on a total of 320 asthmatic patients in Northwest Ethiopia. A simple random sampling technique was employed to select study participants. A pre-tested structured questionnaire and a checklist were used to collect data. Blood samples were collected from asthmatic patients for complete blood count and erythrocyte sedimentation rate determination. Hematological profiles were analyzed by Unicel DxH 800 (Beckman Coulter, Ireland). The erythrocyte sedimentation rate was determined by using the Westergren method. The data were entered into EpiData version 3.0.4 and analyzed with a statistical package for social science version 20 software*.* The bi-variable and multi-variable binary logistic regression models were used to assess the factors associated with hematological abnormalities. A *p* value of less than 0.05 in the multivariable logistic regression analysis was considered statistically significant.

**Results:**

The overall prevalence of neutrophilia, eosinophilia, thrombocytopenia, leukocytosis, and basophilia was 35.3%, 20%, 11.9%, 10.3%, and 4.1%, respectively. Neutrophilia was associated with a lack of physical activity (AOR = 3.25; 95% CI 1.43–7.37) and a history of taking non-asthmatic drugs within the previous three months (AOR = 2.63; 95% CI 1.22–5.65). Being admitted to the emergency department (AOR = 0.27; 95% CI 0.11–5.67) was found to be associated with eosinophilia. In addition, being admitted to the emergency department (AOR = 5.44; 95%CI: 2.6–11.3) was associated with thrombocytopenia.

**Conclusion:**

The current study demonstrated the predominant prevalence of neutrophilia, followed by eosinophilia, among asthma patients. Therefore, hematological abnormalities should be taken into account for proper monitoring and management of asthmatic patients.

**Supplementary Information:**

The online version contains supplementary material available at 10.1186/s12890-022-02020-z.

## Introduction

Asthma is a heterogeneous disease characterized by a chronic inflammatory condition affecting the airways of the lungs. It is a common lung disease that causes narrowing of the airways and results in breathing difficulties [1]. Chronic inflammation in response to triggers (such as allergens and exercise) is associated with excessive airway narrowing (airway hyperresponsiveness) [1, 2]. The diagnosis is made based on the history of characteristic respiratory symptoms, evidence of variable airway limitation, and spirometry [1].

Asthma is a major public health problem. Currently, asthma affects around 339 million people globally [3]. The prevalence of asthma is high in developed nations [4]. However, most asthma-related deaths occur in low and middle-income countries [3, 5]. Although the prevalence of asthma is increasing in less developed countries due to increased urbanization, smoking, and population growth [6].

Different asthma triggers can provoke an allergic reaction, which is associated with the recruitment of pro-inflammatory cells [7]. In the pathogenesis of asthma, eosinophils, basophils, mast cells, macrophages, neutrophils, epithelial cells, and other cellular components play a major role [2]. The pro-inflammatory cells in the airways release specific cytokines such as interleukin (IL)-4, IL-5, IL-9, IL-13, and Granulocyte–Macrophage Colony Stimulating Factor [8]. These cytokines are responsible for the differentiation of eosinophils and the production of immunoglobulin E (IgE) [8]. IgE production induces the release of inflammatory mediators such as histamine and cysteinyl leukotrienes that can directly contract smooth muscles of the airways [8, 9].

The granulocytic parameters have a predictive role in asthma [10]. Predominantly, eosinophils and neutrophils are involved in allergic diseases [11, 12]. Also, basophils are involved in allergic reactions. Basophils express receptors for IgE and release mediators such as histamines and leukotrienes [13].

Neutrophilia and eosinophilia are characteristic features of asthma [14, 15]. IL-8 stimulates the recruitment of neutrophils during exposure to an allergen [16]. As a result, neutrophils can facilitate allergic sensitization and airway inflammation [17]. While eosinophils can help fight against parasitic infection, other infectious agents, and asthma [18]. The recruitment of eosinophils is induced by eotaxin and potent mediators like IL-4 and IL-13 [19].

According to Global Initiative for Asthma (GINA), blood eosinophil count is not used for the diagnosis of asthma [20]. However, it can be used as prognostic biomarker, predict response to therapeutics, and considered as a diagnostic biomarker for defining asthma phenotype. Eosinophilia can be related to the severity of the disease [20, 21]. It is known to be an independent risk factor for asthma exacerbations, emergency department visits, and hospitalization due to asthma [22, 23]. In addition, eosinophilia is related to an increased risk of moderate and severe asthma exacerbations [23, 24]. Eosinophils release lipid mediators and toxic basic proteins that mediate airflow obstruction, bronchial epithelium injury, airway inflammation, and mucous hypersecretion [25, 26]. Several studies have found that eosinophilia can account for up to 40% of asthma patients [27–29].

Platelets play a crucial role in the pathogenesis of asthma [30]. The activation of platelets occurs following exposure to possible asthma triggers [31]. They express receptors that might be necessary for the inflammatory response [30]. Asthmatic patients have low platelet counts after allergen challenges. This is due to pulmonary recruitment and decreased survival of platelets as a result of continuous activation in allergic inflammation [32, 33].

Basophils and mast cells play essential roles in asthma pathogenesis [34]. The mast cells are significant inflammatory cells in the air way smooth muscle that stimulate air way remodeling and mucous hypersecretion [35]. Recent studies showed that mast cell activations can indicate asthma phenotypes [36].

Elevated erythrocyte sedimentation rate (ESR) is typical change in asthma. Basophils, mast cells, and macrophages produce IL-1 and Tumor Necrosis Factor-alpha (TNF-α), which stimulate the liver to produce acute-phase proteins. A rise in acute phase proteins like fibrinogen decreases the net negative charge of the RBC surface and enhances the rouleaux formation. Therefore, rouleaux formation increases the ESR among asthmatic patients [37].

Asthma can affect hematological parameters due to the immune system’s response and changes in hematopoietic physiology. Progression and severity of asthma cause variations in whole blood cell lines [38–40]. Determining the hematological parameters is important for diagnosis, follow-up, prognosis, and management of asthmatic patients [41, 42]. Even though the magnitudes of eosinophilia were reported among asthma patients, the magnitudes of other hematological abnormalities were less reorted. In addition, there was limited data regarding the magnitudes of selected hematological abnormalities and their associated factors among asthma patients in Ethiopia. Therefore, the purpose of this study was to determine the magnitudes of the selected hematological abnormalities and associated factors among asthmatic patients in Northwest Ethiopia.

## Methods and materials

### Study design, area and period

An institution-based cross-sectional study was conducted from March to May 2021 in Northwest Ethiopia at the University of Gondar Comprehensive Specialized Hospital (UGCSH) and Tibebe-Ghion Specialized Hospital (TGSH). UGCSH is located in Northwest Ethiopia, in Amhara Regional State, in Gondar Town. Currently, the hospital provides service to over 7 million people in the catchment area [43]. The hospital has a capacity of 598 beds for inpatients with five disciplines and 12 outpatient departments (OPD). There are over 350 regular follow-ups of asthmatic patients in the chronic OPD of the hospital. On the other hand, TGSH is located in Northwest Ethiopia, in Bahirdar City, about 10 km away from Bahirdar City Square on the way to Adet district. The hospital provides health services for 8 nearby zones in the Amhara regional state.

### Study participants

All adult asthmatic patients attending UGCSH and TGSH were included in the study. Patients who already have chronic diseases like HIV/AIDS, chronic kidney disease, hematological malignancies, and tuberculosis were excluded from this study by reviewing their medical records.

### Sample size determination and sampling technique

#### Sample size determination

A single population proportion formula was used to calculate sample size by [n = (Zα/2)^2^(pq)/d^2^]. By considering the proportion of 50%, 5% marigin error, and 95% confidence interval. The total sample size was calculated as: n = (1.96)^2^ (0.5 × 0.5)/(0.05)^2^, then n_0_ = 384.

However, the estimated number of asthmatic patients in two hospitals per year was 1,200 (data taken from the previous annual report), which is less than 10,000 (N < 10,000). A finite population correction is recommended for this situation. As a result, the sample size was adjusted to n_f_ = n_0_/1 + n_0_/N = 384/1 + 384/1200 = 290.9, approximately n_f_ = 291. Where d = margin of error (5%), n_0_ = initial sample size, N = total number of the target population, and Z α/2 = 1.96 at 95% confidence interval.

Therefore, by adding a 10% non-response rate, the final sample size was 320. The study participants were proportionally allocated from each hospital (200 from UGSH and 120 from TGSH). A lottery-based simple random sampling technique was used to select the study participants.

### Operational definitions

- *Asthma*: any person who has ever received a diagnosis of asthma from a doctor working in internal medicine pulmonary and critical care. Diagnosis was carried out based on a history of variable respiratory symptoms and confirmed variable expiratory airflow limitation according to Global Initiative for Asthma (GINA) guideline [1].
- *Admission to the emergency department*: asthmatic patients who were admitted to the emergency room for health care utilization due to respiratory symptoms [44].
- *The severity of asthma* was classified based on the GINA2019 guideline [45].
- *Stages of asthma* were classified as intermittent, mild persistent, moderate persistent, and severe persistent based on the GINA 2019 guideline [45].
- *A habit of doing physical exercise*: a study subject who has experience of doing physical exercise once per day for at least 20–30 min as a continuous activity [46].
- *Normal value*: Total leukocyte (3.6–10.6 × 10^3^/μl), neutrophil (1.6–5.1 × 10^3^/μl), eosinophil (0.0–0.04 × 10^3^/μl), basophil (0.0–0.2 × 10^3^/μl), platelet (150–450 × 10^3^/μl), and erythrocyte sedimentation rate (for male = 0-15 mm/hr, Female = 0-20 mm/hr) [47].

### Data collection and sample processing

#### Socio-demographic, behavioral and anthropometric measurements

The data related to socio-demographic and behavioral characteristics of study participants were collected with a structured pre-tested questionnaire through a face-to-face interview.

Clinical data of study participants such as duration of a patient living with asthma, family history of asthma, history of asthmatic drug intake before 3 months, chronic disease, severity, stages of asthma, symptoms of asthma, and history of non-asthmatic drug intake before 3 months were collected by reviewing medical records via a checklist. Anthropometric variables such as height (in meters) and weight (in kilograms) were measured by clinical nurses. Then, body mass index (BMI) was computed as weight in kilograms divided by height in meters squared. After trained clinical nurses had completed data collection through interviews, reviewing medical records, and anthropometric measurements, the study participants were sent to the laboratory room where a blood sample was drawn.

#### Hematological analysis

A six-milliliter venous blood specimen was collected from asthmatic patients by the trained laboratory technician through venipuncture of the superficial veins of the antecubital fossa. After blood collection, 3 ml of blood was transferred into the tube containing the Ethylenediaminetetraacetic acid (EDTA) test tube and gently mixed. The left-over blood (3 ml) was dispensed into a tube containing 3.1 g/L trisodium citrate in a 1:4 ratio (1 part of trisodium citrate with 4 parts of blood) for ESR value determination.

Five differential Unicel DxH 800 Coulter cellular analysis systems (Beckman Coulter, Ireland) were used to analyze the Complete Blood Count (CBC). This automated hematological analyzer uses principles of impedance, spectrophotometry, and VCS technology. Peripheral blood morphology assessment was done to confirm flags by the automated hematology analyzer and to check the quality of automated hematology analyzer. A thin blood smear was prepared from leftover blood from CBC. Then the air-dried smear was stained with Wright stain, and the blood cell morphological features were assessed with a 100× oil immersion by laboratory technologists.

In a 3:4 ratio, whole blood was diluted with a 3.1 g/L trisodium citrate anticoagulant in a 1:4 ratio. Then a trisodium citrate diluted blood sample was left for one hour on the Westergren method of ESR determination set up. After one hour, the height of plasma was measured and reported in millimeters per hour (mm/h).

#### Parasitological examination

A pea-sized (1 g) fresh stool specimen was obtained from every study participant in clean and labeled leak-proof stool containers. A Formol-ether concentration technique was performed to concentrate intestinal parasites. From sediment, a wet mount was prepared and examined under a microscope with 10× and 40× objectives. Laboratory tests like CBC, peripheral morphology, ESR, and stool examination were performed in the UGCSH and TGSH laboratories and recorded for every study participant using a form designed for laboratory result registration.

### Data quality assurance and management

The questionnaire was prepared in the English version and translated to the local language (Amharic) and retranslated back to the English version to check the accuracy and consistency. The questionnaire was pretested on 5% of asthmatic patients at Felege Hiwot Comprehensive Specialized Hospital, and the questionnaire was modified. The training was given to the data collectors before starting data collection to reduce technical and observer bias.

Pre-analytical, analytical, and post-analytical phases of quality assurance were maintained to assure the quality of data. Quality control (QC) for working equipment and reagents was ensured by using standard controls. Low, normal, and high QC materials were run to check the functioning of the hematological analyzer. SOPs and manufacturer’s instructions were strictly followed during sample collection and laboratory procedures. The questionnaire was checked for consistency, clarity, and completeness. The results of every test were properly recorded, reviewed, and transcribed.

### Data processing and statistical analysis

The collected data was entered into Epi-data software (Version: 3.0.4), then cleaned and exported into a Statistical Package for Social Science version 20 (IBM Corp., Armonk, NY, USA) for analysis. Descriptive statistics such as frequencies and percentages were used to summarize the data. The Kolmogorov–Smirnov test and histogram were used to check the normal distribution of data. The values of selected hematological parameters were presented with median and interquartile range (IQR). Non-parametric mann–Whitney U tes was used to compare median difference in hematological parameters among groups. The data was presented using tables, charts, and graphs. Bivariable and multivariable logistic regression analysis was done. Crude odds ratio (COR) and adjusted odds ratio (AOR) with a 95% confidence interval (CI) were used to observe the strength of the association between the predictors and the outcome. The backward selection method was used to select the important variables. The variables with a *p* value of < 0.25 in the bivariable logistic regression analysis were fitted into the multivariable logistic regression analysis. The model of fitness was checked by Hosmer and Lemeshow’s goodness-of-fit statistic. A *p* value of less than 0.05 was considered statistically significant in any condition.

### Ethical consideration

Ethical clearance was obtained from the ethical review committee of the School of Biomedical and Laboratory Science, College of Medicine and Health Sciences, University of Gondar, with letter reference number SBMLS-2748/2021. A support letter from the Department of Hematology and Immunohematology was submitted to UGCSH and TGSH. Permission was obtained from the UGCSH and TGSH directors.

Informed written consent was obtained from each study participant after the objective of the study was explained. For illiterate study subjects, informed consent was obtained after information was read to the study participants by the data collectors. Then a fingerprint signature was taken from each illiterate study participant. To maintain confidentiality, the study participants were identified by using codes rather than individual identifiers, and unauthorized persons were not able to access the collected data. The results and information of the study participants were kept confidential. Study participants with hematological abnormalities were linked to the UGCSH and TGSH chest clinic departments for proper management. All methods were performed in accordance with the relevant guidelines and regulations (Declaration of Helsinki).

## Results

### Socio-demographic characteristics of the study participants

A total of three hundred twenty (n = 320) adult asthmatic patients were enrolled in this study. There were 178 (55.6%) females among them. The age of study participants ranged from 18 to 65 years, with a median and interquartile range (IQR) of 50 (38–60) years. 206 (64.4%) and 204 (63.8%) of the participants were older than 40 years old and living in urban areas, respectively. Regarding the educational status of study participants, 140 (43.8%) were unable to read and write. Among study participants, 109 (34.17%) were housewives, and 213 (66.6%) were married (Additional file 1).

#### Behavioral, clinical characteristics, and BMI of the study participants

Of the study participants, 5 (1.6%) were current cigarette smokers, 54 (16.9%) had a habit of doing physical exercise, and 75 (23.4%) had the habit of drinking alcohol. Of those who had a habit of drinking alcohol, about 51 (15.9%) were frequent drinkers. Among study participants, 56 (17.5%) had a high BMI (Table 1).

**Table 1:** Behavioral, clinical characteristics, and BMI of asthmatic patients in Northwest Ethiopia, 2021 (n = 320)

Characteristics	Frequency	Percentage %
Cigarette smoking		
Non smoker	315	98.4
Smoker	5	1.6
Physical exercise habit		
Yes	54	16.9
No	266	83.1
Alcohol drinking habit		
Yes	75	23.4
No	245	76.6
Frequency of alcohol drinking		
Frequent drinker	51	15.9
Social drinker	24	7.5
BMI		
Underweight	26	8.1
Normal weight	238	74.4
Over weight	56	17.5
Family history of asthma		
Yes	107	33.4
No	213	66.6
Duration of living with asthma (in years)		
≤ 5	127	39.7
6–10	97	30.3
> 10	96	30
Severity of asthma		
Mild	34	10.6
Moderate	234	73.1
Severe	52	16.2
Stages of asthma		
Intermittent	40	12.5
Mild persistent	25	7.8
Moderate persistent	204	63.8
Severe persistent	51	15.9
Asthmatic drug intake before 3 months		
Yes	262	81.9
No	58	18.1
Non -asthmatic drugs intake before 3 months		
Yes	96	30
No	224	70
Symptoms of asthma		
Shortness of breath	119	37.2
Cough	105	32.8
Wheezing	15	4.7
Runny nose	24	7.5
2 or more symptoms	37	11.6
Other^a^	20	6.2
Chronic disease		
Yes	92	28.8
No	228	71.3
Types of chronic disease		
HTN	62	67.4
DM	14	15.2
Other^b^	16	17.4
Admission to the ED		
Yes	89	27.8
No	231	72.2

BMI, Body Mass Index; ED, emergency department; HTN, hypertension; DM, diabetes mellitus

Other^a^ (symptoms other than asthma such as, headache, joint pain, waist pain), other^b^ (include epilepsy, dyslipidemia, and rheumatoid arthritis

About 107 (33.4%) of study participants had a family history of asthma, 127 (39.7%) had been living with asthma for less than five years, 234 (73.1%) had asthma with moderate severity, and 204 (63.8%) had a moderate persistent stage of asthma. Regarding symptoms of asthma, 119 (37.2%) of study participants experienced shortness of breath, and 105 (32.8%) had a cough. About 261 (81.6%) of the study participants had taken asthmatic drugs in the previous three months. Among the study participants, about 89 (27.8%) were admitted to the emergency department (Table 1).

**Fig. 1:**
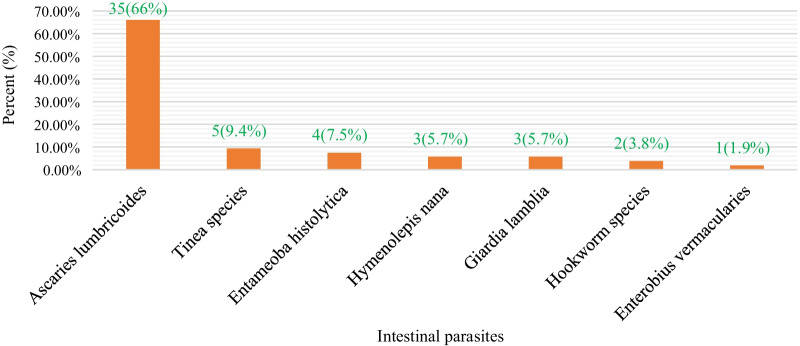
A bar chart shows the types of intestinal parasites among asthmatic patients

Of the study participants, about 47 (14.7%) were infected with one or more intestinal parasites. Among those who had a parasitic infection, Ascaris *lumbricoides* was found in 35 (66%), followed by Taenia species 5 (9.4%) (Fig. 1).

### Magnitudes of hematological abnormalities

The majority of study participants had neutrophilia, 113 (35.3%, 95% CI 30.1–40.8%), followed by eosinophilia, 64 (20%, 95% CI 15.8–24.8%). About 38 (11.9%, 95% CI 8.4–15.6%) had thrombocytopenia, leukocytosis, 33 (10%, 95% CI 6.9–14.1), and basophilia, 13 (4%, 95% CI 2.2–6.3).. The median and IQR values of the selected hematological parameters are WBC, 6.6 (4.8–8.75), neutrophil count, 3.94 (2.4–5.87), eosinophil count, 0.2 (0.07–0.4), basophil count, 0.03 (0.00–0.1), platelet, 229 (183–287), and ESR, 20 (5–43). In addition, an increased ESR value was observed among 163 (50.9%) of the study participants.

### Comparison of hematological parameters among different groups

Since the data was not normally distributed, a non-parametric Man-Whitney U test was done to compare the median difference in hematological parameters among different groups like intestinal parasitic infection, systemic steroid use, and ICS use. Based on this analysis, the absolute eosinophil count was significantly higher among asthmatic patients who had a parasitic infection than those who did not have an intestinal parasite (*p* < 0.04). Also, the eosinophil count was significantly lower among asthmatic patients who had taken ICS than among patients who had not taken ICS (*p* < 0.03). In addition, the absolute basophil count showed a statistically significant median difference among patients who used SC and those who had not taken SC (*p* < 0.05). However, platelet and absolute neutrophil counts did not show a statistically significant median (IQR) difference among any of the groups (Table 2).

**Table 2:** Comparision of hematological parameters among different variable groups (Mann–Whitney U test)

Hematological parameter	IP			SC use			ICS		
	Yes	No	*p* value	Yes	No	*p* value	Yes	No	*p* value
EOS# median (IQR)	0.3 (0.09–0.4)	0.2 (0.06–0.4)	0.04*	0.11 (0.037–0.34)	0.2 (0.06–0.4)	0.53	0.1 (0.03–0.3)	0.3 (0.1–0.49)	0.000*
NEU# median (IQR)	3.75 (2.75–5.67)	3.73 (2.3–5.59)	0.73	4.45 (2.37–6)	3.7 (2.3–5.56)	0.46	3.66 (2.23–5.55)	3.9 (2.3–5.63)	0.786
BAS# median (IQR)	0.06 (0.0–0.1)	0.0 (0.0–0.1)	0.11	0.0 (0.0–0.015)	0.03 (0.0–0.1)	0.038*	0.05 (0.0–0.1)	0.0 (0.0–0.1)	0.091

IP, intestinal parasitic infection; SC, systemic corticosteroid; ICS, inhaled corticosteroids; IQR, inter quartile range; EOS#, eosinophil count; NEU#, neutrophil count; BAS#, basophil count

^*^shows statistically significant association

### Factors associated with neutrophilia

In bivariable logistic regression analysis, physical exercise habits, asthmatic drug intake before three months, and admission to the emergency department were shown to have an association with neutrophilia. Multivariable logistic regression analyses revealed that lack of physical exercise (AOR = 3.25; 95% CI 1.43–7.37) and taking drugs other than asthmatic drugs before three months (AOR = 2.63; 95% CI 1.22–5.67) were found to have a statistically significant association with neutrophilia (Table 3).

**Table 3:** Bivariable and multivariable logistic regression analysis of factors associated with neutrophilia among asthmatic patients in Northwest Ethiopia (n = 320)

Variables	Category	Neutrophilia		COR (95% CI)	*p* value	AOR (95% CI)
		Yes N (%)	No N (%)			
Age	≤ 40	46 (40.3%)	68 (59.7%)	1^a^		1^a^
	> 40	67 (32.5%)	139 (67.5%)	0.71 (0.44–1.14)	0.161	0.64 (0.36–1.13)
Gender	Male	55 (38.7%)	87 (61.3%)	1^a^	0.25	–
	Female	58 (32.6%)	120 (67.4%)	1.31 (0.82–2.07)		
Residence	Urban	67 (32.8%)	137 (67.2%)	0.74 (0.46–1.19)	0.221	0.98 (0.57–1.7)
	Rural	46 (39.6%)	70 (60.4%)	1^a^		1^a^
Educational status	Unable to read and write	55 (39.3%)	85 (60.7%)	1.24 (0.67–2.50)	0.557	–
	Primary school	25 (33.8%)	49 (66.2%)	1.02 (0.48–2.14)		–
	Secondary school	15 (28.8%)	37 (71.2%)	0.81 (0.35–1.85)		–
	College and above	18 (33.3%)	36 (66.7%)	1^a^		
Occupation	House wife	35 (32.1%)	74 (67.9%)	0.83 (0.32–2.16)	0.561	–
	Farmer	27 (41.5%)	38 (58.5%)	1.24 (0.46–3.38)		–
	Employer	17 (29.3%)	41 (70.7%)	0.73 (0.26–2.05)		–
	Private worker	26 (39.4%)	40 (60.6%)	1.14 (0.42–3.09)		–
	Others	8 (36.4%)	14 (63.6%)	1^a^		
Family size	≤ 4	48 (32%)	102 (68%)	1^a^	0.244	1^a^
	5–8	54 (36.5%)	94 (63.5%)	1.22 (0.76–1.97)		1.17 (0.69–2)
	> 8	11 (50%)	11 (50%)	2.12 (0.86–5.24)		1.60 (0.58–4.44)
Physical exercise habit	Yes	9 (16.7%)	45 (83.3%)	1^a^		1^a^
	No	104 (39.1%)	162 (60.9%)	3.21 (1.51–6.84)	0.033	3.25 (1.43–7.37)*
Alcohol drinking habit	Yes	28 (37.3%)	47 (62.7%)	1.12 (0.66–1.92)	0.676	–
	No	85 (34.7%)	160 (65.3%)	1^a^		
Duration of patient living with asthma	≤ 5	52 (40.9%)	75 (59.1%)	1^a^	0.142	1^a^
	6–10	34 (35%)	63 (65%)	0.78 (0.45–1.34)		1.15 (0.62–2.15)
	> 10	27 (39%)	69 (61%)	0.56 (0.32–1.0)		0.95 (0.48–1.89)
Family history of asthma	Yes	32 (29.9%)	75 (70.1%)	0.69 (0.42–1.14)	0.152	0.75 (0.42–1.31)
	No	81 (38%)	132 (62%)	1^a^		1^a^
Severity of asthma	Mild	17 (50%)	17 (50%)	1^a^		1^a^
	Moderate	80 (34.2%)	154 (65.8%)	0.52 (0.25–1.07)	0.156	0.52 (0.23–1.15)
	Severe	16 (30.8%)	36 (69.2%)	0.44 (0.18–1.09)		0.46 (0.17–1.25)
Stages of asthma	Intermittent	15 (37.5%)	25 (62.5%)	1^a^		1^a^
	Mild persistent	7 (28%)	18 (72%)	0.65 (0.22–1.91)	0.466	–
	Moderate persistent	77 (37.7%)	127 (62.3%)	1.01 (0.50–2.03)		–
	Severe persistent	14 (27.4%)	37 (72.6%)	0.63 (0.26–1.53)		–
Asthmatic drugs intake before 3 months	Yes	85 (32.4%)	177 (67.6%)	1.94 (1.09–3.46)	0.024	0.64 (0.32–1.28)
	No	28 (48.3%)	30 (51.7%)	1^a^		1^a^
SC use	Yes	8 (44.4%)	10 (55.6%)	1.73 (0.66–4.57)	0.265	–
	No	77 (31.6%)	167 (68.4%)	1^a^		
Non-asthmatic drug intake before 3 months	Yes	40 (41.7%)	56 (58.3%)	1.48 (0.9–2.42)	0.12	2.63 (1.22–5.67)*
	No	73 (32.6%)	151 (67.4%)	1^a^		1^a^
Chronic disease	Yes	30 (32.6%)	62 (67.4%)	1.48 (0.90–2.42)	0.12	0.59 (0.26–1.35)
	No	83 (36.4%)	145 (63.6%)	1^a^		1^a^
Admission to the ED	Yes	42 (47.2%)	47 (52.8%)	2.01 (1.22–3.32)	0.006	1.22 (0.63–2.39)
	No	71 (30.7)	160 (69.3%)	1^a^		1^a^
Intestinal parasitic infection	Yes	13 (27.7%)	34 (72.3%)	0.66 (0.33–1.31)	0.237	0.75 (0.36–1.58)
	No	100 (36.6%)	173 (63.4%)	1^a^		1^a^
BMI	Under weight	11 (42.3%)	15 (57.7%)	1.32 (0.58–3.00)	0.557	–
	Normal weight	85 (35.7%)	153 (64.3%)	1^a^		
	Over weight	17 (30.4%)	39 (69.6%)	0.78 (0.42–1.47)		–

IP, intestinal parasite; BMI, body mass index; COR, crude odds ratio; AOR, adjusted odds ratio, ED, emergency department

^*^Shows significant association

1^a^ = shows reference group

### Factors associated with eosinophilia

In bivariable logistic regression analysis, alcohol drinking habits, educational status, SC use, and admission to the emergency department were associated with eosinophilia. In contrast, being admitted to the ED (AOR = 0.17; 95% CI 0.05–0.62) and SC use (AOR = 0.27; 95% CI 0.08–0.89) only showed a statistically significant association with eosinophilia in multivariable logistic regression analysis (Table 4).

**Table 4:** Bivariable and multivariable logistic regression analysis of factors associated with eosinophilia among asthmatic patients in Northwest Ethiopia (n = 320)

Variables	Category	Eosinophilia		COR (95% CI)	*p* value	AOR (95% CI)
		Yes N (%)	No N (%)			
Age	≤ 40	27 (23.7%)	87 (76.3%)	1^a^		1^a^
	> 40	37 (18%)	169 (82%)	0.70 (0.40–1.23)	0.222	0.52 (0.26–1.03)
Gender	Male	35 (10.9%)	107 (33.4%)	1^a^	0.065	1^a^
	Female	29 (16.3%)	149 (83.7%)	1.68 (0.97–2.92)		0.47 (0.33–1.04)
Residence	Urban	39 (19.1%)	165 (80.9%)	0.86 (0.49–1.51)	0.601	–
	Rural	25 (21.5%)	91 (78.5%)	1^a^		
Marital status	Single	34 (79.1%)	9 (20.9%)	1^a^	0.82	
	Married	169 (79.4%)	44 (20.6%)	0.98 (0.44–2.20)		–
	Separated	53 (82.8%)	11 (17.3%)	0.78 (0.29–2.09)		–
Educational status	Unable to read and write	30 (21.4%)	110 (78.6%)	1.57 (0.67–3.68)	0.06	2.04 (0.69–6.04)
	Primary school	21 (28.4%)	53 (71.6%)	2.28 (0.92–5.63)		2.4 (0.78–4.85)
	Secondary school	5 (9.6%)	47 (90.4%)	0.61 (0.19–2.01)		0.73 (0.2–2.77)
	College and above	8 (14.8%)	46 (85.2%)	1^a^		1^a^
Alcohol drinking habit	Yes	23 (30.7%)	52 (69.3%)	2.20 (1.214–3.99)	0.009	1.9 (0.99–4.61)
	No	41 (16.7%)	204 (83.3%)	1^a^		1^a^
Physical exercise habit	Yes	8 (14.8%)	46 (85.2%)	1^a^		
	No	56 (21%)	210 (79%)	1.53 (0.68–3.43)	0.299	–
Family history of asthma	Yes	24 (22.4%)	83 (77.6%)	1.25 (0.71–2.21)	0.442	–
	No	40 (18.8%)	173 (81.2%)	1^a^		
Duration of patient living with asthma	≤ 5	26 (20.5%)	101 (79.5%)	1^a^		
	6–10	21 (21.7%)	76 (78.3%)	1.07 (0.56–2.05)	0.78	–
	> 10	17 (17.7%)	79 (82.3%)	0 .84 (0.42–1.65)		–
Severity of asthma	Mild	5 (14.7%)	29 (85.3%)	1^a^		
	Moderate	47 (20.1%)	187 (79.9%)	1.46 (0.53–3.97)	0.639	–
	Severe	12 (23.1%)	40 (76.9%)	1.74 (0.55–5.48)		–
Asthmatic drugs intake before 3 months	Yes	55 (21%)	207 (79%)	1.45 (0.67–3.13)	0.348	–
	No	9 (15.5%)	49 (74.5%)	1^a^		
Non-asthmatic drug intake before 3 months	Yes	21 (22%)	75 (78%)	1.18 (0.65–2.12)	0.583	–
	No	43 (19.2%)	181 (80.8%)	1^a^		
Chronic disease	Yes	20 (21.7%)	72 (78.3%)	1.10 (0.60–2.01)	0.756	–
	No	44 (19.3%)	184 (80.7%)	1^a^		
Admission to the ED	Yes	7 (7.9%)	82 (92.1%)	0.26 (0.11–0.60)	0.001	0.17 (0.05–0.62)*
	No	57 (24.7%)	174 (75.3%)	1^a^		1^a^
SC use	Yes	8 (44.4%)	10 (55.6%)	0.298 (0.11–0.78)	0.016	0.27 (0.08–0.89)*
	No	47 (19.3%)	197 (80.7%)	1^a^		1^a^
Intestinal parasitic infection	Yes	9 (19.1%)	38 (80.9%)	0.94 (0.43–2.06)	0.875	–
	No	55 (20.1%)	218 (79.9%)	1^a^		
BMI	Under weight	5 (19.2%)	21 (80.8%)	0.92 (0.33–2.56)	0.895	–
	Normal weight	49 (20.6%)	189 (79.4%)	1^a^		
	Over weight	10 (17.9%)	46 (82.1%)	0.84 (0.39–1.78)		–

SC, systemic corticosteroids; DM, diabetes mellitus; HTN, hypertension; COR, crude odds ratio; AOR, adjusted odds ratio; BMI, body mass index; ED, emergency department

^*^Indicates statistically significant

1^a^ = reference group

### Factors associated with thrombocytopenia

In bivariable logistic regression analysis, residence, alcohol drinking habit, duration of a patient living with asthma, SC use, and admission to the emergency department were associated with thrombocytopenia. In multivariable binary logistic regression analysis, being admitted to the emergency department (AOR = 5.44; 95% CI 2.6–11.3) and living in an urban area (AOR = 2.79; 95% CI 1.01–7.72) showed a statistically significant association with thrombocytopenia (Table 5).

**Table 5:** Bivariable and multivariable logistic regression analysis of factors associated with thrombocytopenia among asthmatic patients in Northwest Ethiopia (n = 320)

Variables	Category	Thrombocytopenia		COR (95% CI)	*p* value	AOR (95% CI)
		Yes N (%)	No N (%)			
Age	≤ 40	9 (7.9%)	105 (92.1%)	1^a^	0.106	1^a^
	> 40	29 (14.1%)	177 (85.9%)	1.91 (0.87–4.19)		2.13 (0.93–4.88)
Gender	Male	18 (12.7%)	124 (87.3%)	1^a^	0.693	–
	Female	20 (11.2%)	158 (88.8%)	1.15 (0.58–2.26)		
Residence	Urban	32 (15.7%)	172 (84.3%)	3.41 (1.38–8.42)	0.008	2.79 (1.01–7.72)*
	Rural	6 (5.2%)	110 (94.8%)	1^a^		1^a^
Physical exercise habit	Yes	6 (11.1%)	48 (88.9%)	1^a^		
	No	32 (12%)	234 (88%)	1.09 (0.43–2.76)	0.849	–
Family history of asthma	Yes	10 (9.3%)	97 (90.7%)	0.68 (0.32–1.46)	0.324	–
	No	28 (13.1%)	185 (86.9%)	1^a^		
Duration of patient living with asthma	≤ 5	22 (17.3%)	105 (82.7%)	1^a^	0.053	1^a^
	6–10	9 (9.3%)	88 (90.7%)	0.49 (0.21–1.11)		0.48 (0.16–1.4)
	> 10	7 (7.3%)	89 (92.7%	0.37 (0.15–0.92)		0.61 (0.2–1.8)
Asthmatic drugs intake before 3 months	Yes	27 (10.3%)	235 (89.7%)	0.49 (0.23–1.06)	0.069	1.1 (0.43–2.68)
	No	11 (18.9%)	47 (81%)	1^a^		1^a^
Non-asthmatic drug intake before 3 months	Yes	9 (9.4%)	87 (90.6%)	0.7 (0.32–1.53)	0.367	–
	No	29 (12.9%)	195 (87.1%)	1^a^		
SC use	Yes	5 (27.8%)	13 (72.2%)	3.88 (1.27–9.1)	0.018	3.3 (0.9–10.34)
	No	22 (9%)	222 (91%)	1^a^		1^a^
Chronic disease	Yes	12 (13%)	80 (87%)	1.21 (0.58–2.51)	0.614	–
	No	26 (11.4%)	202 (88.6%)	1^a^		
Admission to the ED	Yes	23 (25.8%)	66 (74.2%)	5.02 (2.48–10.17)	0.0001	5.44 (2.6–11.3)*
	No	15 (6.5%)	216 (93.5%)	1^a^		1^a^

COR, crude odds ratio; AOR, adjusted odds ratio; ED, emergency department; SC, systemic corticosteroids

^*^Indicates significant association

1^a^ = reference group

## Discussion

An institution-based cross-sectional study was conducted to determine the selected hematological abnormalities and their associated factors among asthmatic patients. In this study, we assessed the magnitude and factors associated with hematological abnormalities.

In the present study, leukocytosis was observed in 10.3% (95% CI 7.2–14.2%) of asthmatic patients. Even though the magnitude of leukocytosis was not reported, different studies indicated that leukocytosis is a common feature among asthmatic patients [21, 41, 48]. Previous studies in Turkey [42], Pakistan [40], India [48], Iraq [21], and Ethiopia [41] showed that mean values of WBC count were higher in asthma patients when compared to their counterparts. The possible reason for the elevated leukocyte count in asthmatic patients might be due to the release of inflammatory cytokines and the effect of drugs [49].

Based on the findings of this study, the most common hematological abnormality was neutrophilia, with a prevalence of 35.3% (95% CI 30.1–40.8%). Similar studies conducted in Turkey [42], Pakistan [40], India [48] and Ethiopia [41] showed that the mean value of neutrophil count was higher among asthma patients than healthy controls, but the percentage was not reported. The possible reason for an increase in neutrophils might be associated with cytokine production, particularly IL-8. IL-8 is found to be increased in asthmatic patients [50]. It is a potent neutrophil recruiting factor by binding to the receptors [11, 51].

Unlike eosinophils, neutrophils are the first cell type to enter the lung following an allergen challenge [16]. Moreover, neutrophils facilitate allergic sensitization and airway inflammation [17]. Another possible explanation might be due to variation on age of study participants. In this study majority of study participants were greater than 50 years old (82 (25.6%) were greater than 50 years old versus 87 (27.2%) were greater than 60 years old). Neutrophils are known to be increased in elderly asthma patients [52]. Although blood neutrophil count is not a biomarker for the diagnosis of asthma, the EGEA study suggested that persistent blood neutrophilia (> 5000 cells/μl) could be a prognostic biomarker, associated with poor system control and increased exacerbation [10].

In the present study, the prevalence of eosinophilia was 20% (95% CI 15.8%‒24.8%). This finding is in line with the study conducted in the USA (18.5%) [53], the UK (16%) [54], and a cross-sectional survey of the US general population (18%) [55]. But the finding was lower than a pilot study conducted in the US (40%) [27], Japan (34%) [56], Canada (41%) [28], Brazil (40%) [29], Mexico (37.7%) [57], and the UK (43%) [58]. The possible explanation for the difference might be due to variation in study participants, socio-demographic characteristics, and sample size. Although the circulating eosinophil levels are elevated in patients with allergic conditions, in this circumstance, eosinophil recruitment is induced by IL-5 and eotaxin [19, 59].

Moreover, eosinophilia is considered a prognostic marker and can be associated with frequent asthma exacerbations [21, 60]. High eosinophil counts in the blood can be linked to an increase odds of asthma exacerbation [23]. Recent studies reported that patients with a blood eosinophil count greater than 400 cells/μl experienced severe asthma exacerbation, acute respiratory conditions, and a risk for airflow obstruction among asthma patients [54, 61].

Thrombocytopenia was observed in 11.9% (95% CI 8.5–15.9%) of asthmatic patients. A similar study was done in Jimma and showed that mean values of platelet count were lower in asthma patients than in healthy controls [41] but the percentage was not reported. The possible reason for the decreased peripheral platelets among asthmatic patients might be due to localized recruitment of platelets to the lungs and the presence of leukocyte platelet complexes in circulation [33].

Basophilia was observed in 4.1% of asthmatic patients. This finding was lower than a similar study done in Mexico (37.7%) [57]. The possible reason for the discrepancy might be due to the difference in the reference range used (≥ 110 cells/μl versus > 200 cells/μl) and the sample size used. Furthermore, basophils are known to be actively involved in allergic disorders such as asthma [13, 48].

Increased ESR values were observed in 50.9% (95% CI 45.3–56.5%) of asthmatic patients. Although the percentage was not reported, similar studies were done in India [48] and Jimma [41] that indicated that the mean values of ESR were higher among asthma patients when compared to their counterparts. This might be due to the production of IL-1 and TNF-α by mast cells, basophils, and macrophages, which stimulate the liver to produce acute-phase proteins. As a result, a rise in acute-phase proteins like fibrinogen increases the positive charge on the RBC surface and enhances the stacking of RBCs. An increase in rouleaux formation leads to increased ESR [37].

Based on the findings of this study, the study participants who were diagnosed with intestinal parasitic infection had significantly higher absolute eosinophil count than those who were not infected with intestinal parasitic infection. This finding was supported by different studies [62, 63]. The parasitic infections are characterized by up regulation of TH2 cytokins. As a result, these cytokines increase infiltration of eosinophils, survival, and anti-parasitic activity [64].

This study also determined the association between hematological abnormalities and their associated factors among asthma patients. We have done a multivariable logistic regression analysis for leukocytosis. But any of the variables did not show a statistically significant association with leukocytosis.

Asthmatic patients who do not have the habit of doing physical exercise were three times more likely to develop neutrophilia when compared to patients doing physical exercise. This might be due to the fact that poor exercise worsens asthma outcomes. A worsened asthma state causes frequent asthma attacks and increases asthma exacerbation [65]. In addition, Huovinen E et al*.* showed that physical activity had a protective effect on asthma [66].

Study participants who had taken non-asthmatic drugs before three months were 3 times more likely to develop neutrophilia than patients who had not taken non-asthmatic drugs. This might be due to the effects of drugs. Different drugs (like glucocorticoids) can cause neutrophilia by inducing the release of neutrophils from the bone marrow [67].

The study subjects who were admitted to the emergency department were less likely to develop eosinophilia. This might be due to patients admitted to the emergency department being treated with high doses of ICS (according to GINA guidelines) [1]. ICS is known to lower the eosinophil count due to its anti-inflammatory effect [68–70]. ICS reduces inflammation in the airway epithelial cells by inhibiting transcription of genes encoding proteins (such as IL-4, IL-5, IL-13, and TNF-α), reducing recruitment of inflammatory cells through inhibition of chemokines and adhesion molecules, and decreasing the survival of inflammatory cells (such as eosinophils, T-lymphocytes, and mast cells) [70]. Moreover, eosinophil recruitment is reduced due to low cytokine production, especially IL-5 [71].

Asthmatic patients who had taken SC were less likely to develop eosinophilia. This finding was supported by different studies [72–74]. The possible explanation might be due to the fact SC has an anti-inflammatory effect and known to reduce eosinophil count [73]. In asthma patients, the therapeutic SC dose induce reduction in blood eosinophil level [72].

Study participants who were admitted to the emergency department were 5 times more likely to develop thrombocytopenia. The possible explanation may be due to patients admitted to the emergency department having more severe asthma attacks. When inflammation increases (severe asthma), platelets become activated and are recruited to the pulmonary [33].

Study participants who lived in urban areas were 2.79 times more likely to develop thrombocytopenia. This might be due to the fact that the outdoor air of urban areas is polluted due to high traffic and industry-related emissions that can increase the risk of asthmatic attacks. Thus, patients who live in urban areas might have more asthma attacks than patients living in rural areas. An increase in the severity of asthma can be linked with peripheral thrombocytopenia.

This study had some limitations. The cross-sectional nature of the study makes it difficult to establish a cause-effect relationship between hematological changes and associated factors (it is a temporal relationship). Another limitation is that important cytokines that mediate pro-inflammatory cell recruitment (like IL-4, IL-5, and IL-8) were not analyzed due to a lack of testing materials.

## Conclusion and recommendations

In the present study, neutrophilia was the most frequent hematological abnormality identified, followed by eosinophilia and thrombocytopenia. The hematological abnormalities were common among asthmatic patients. Therefore, considering selected hematological abnormalities should be important for the proper monitoring and management of asthmatic patients.

No habit of doing physical exercise and taking non-asthmatic drugs before three months was associated with neutrophilia. On the other hand, being admitted to the emergency department was associated with eosinophilia and thrombocytopenia. Checking the hematological profiles of asthmatic patients would be important for reducing the risk of emergency department admission and further complications. Further longitudinal studies will be encouraged to see the effect of systemic corticosteroid use and other drugs on hematological parameters among asthma patients.

## Supplementary Information

**Additional file 1**. Socio-demographic characteristics of asthmatic patients in Northwest Ethiopia, 2021 (n = 320).

## Data Availability

All the data on which the conclusions of this manuscript were drawn are available from the corresponding author. So that anyone who needs the data can get it upon reasonable request.

## Abbreviations

AOR: Adjusted odds ratio
BMI: Body mass index
CBC: Complete blood count
CI: Confidence interval
COR: Crude odds ratio
EDTA: Ethylene diamine tetra acetic acid
ESR: Erythrocyte sedimentation rate
SC: Systemic corticosteroids
ICS: Inhaled corticosteroids
IgE: Immunoglobulin E
IL: Interleukin
GINA: Global Initiative for Asthma
OPD: Outpatient departments
QC: Quality control
RBC: Red blood cell
SOP: Standard Operating Procedure
SPSS: Statistical Package for Social Science
TGSH: Tibebe-Ghion Specialized Hospital
TNF-α: Tumor necrosis factor α
UGCSH: University of Gondar Comprehensive Specialized hospital
WBC: White blood cell
