# Supplementary material for: Selected hematological abnormalities and their associated factors among asthmatic patients in Northwest Ethiopia: a cross-sectional study
Source: BMC Pulm Med. 2022 Jun 13;22:228. doi: 10.1186/s12890-022-02020-z (PMC9190135; doi:10.1186/s12890-022-02020-z)
Supplement: Supplementary file 1 — Additional file 1. Socio-demographic characteristics of asthmatic patients in Northwest Ethiopia, 2021 (n = 320). [file 12890_2022_2020_MOESM1_ESM.docx]

Socio-demographic characteristics of asthmatic patients in Northwest Ethiopia, 2021 (n = 320).

| **Characteristics** | | **Frequency** | **Percentage** |
| --- | --- | --- | --- |
| Age in years ^#^ | ≤40 | 114 | 36.6% |
|  | >40 | 206 | 64.4% |
| Gender | Male | 142 | 44.4% |
|  | Female | 178 | 55.6% |
| Residence | Urban | 204 | 63.8% |
|  | Rural | 116 | 36.2% |
| Marital status | Single | 43 | 13.4% |
|  | Married | 213 | 66.6% |
|  | Separated | 64 | 20% |
| Educational status | Unable to read and write | 140 | 43.8% |
|  | Primary school | 74 | 23.1% |
|  | Secondary school | 52 | 16.2% |
|  | College and above | 54 | 16.9% |
| Occupation | House wife | 109 | 34.1% |
|  | Farmer | 65 | 20.3% |
|  | Employer | 58 | 18.1% |
|  | Private worker | 66 | 20.6% |
|  | Others ^a^ | 22 | 6.9% |
| Family size | ≤4 | 150 | 46.9% |
|  | 5-8 | 148 | 46.2% |
|  | >8 | 22 | 6.9% |

**Note:** **others ^a^** (no job including students), age in years**^#^** adopted from Global Initiative for Asthma (GINA) guideline for asthma management and prevention 2021 (1).
